# Supplementary material for: Visuoinertial and visual feedback in online steering control
Source: PLoS Comput Biol. 2025 Aug 11;21(8):e1012659. doi: 10.1371/journal.pcbi.1012659 (PMC12360656; doi:10.1371/journal.pcbi.1012659)
Supplement: S1 Appendix — Discussion of frequency response properties of the model and its variants. (PDF) [file pcbi.1012659.s001.pdf]

Supporting information

Appendix

Modelling details

As indicated in the main text our model consists of a Linear Quadratic Gaussian System [1]. This is concisely expressed in terms of the system, sensory feedback and a cost function,

$$\text{System } : \mathbf{x}_{t+1} = A\mathbf{x}_t + B\mathbf{u}_t + V\boldsymbol{\eta}_t + V_\epsilon \boldsymbol{\epsilon}_t \tag{A.1}$$

$$\text{Feedback } : \mathbf{y}_t = H\mathbf{x}_t + G\boldsymbol{\omega}_t \tag{A.2}$$

$$\text{Cost } : J = \frac{1}{2}\mathbb{E}[\sum_{t=0}^{\infty}(\mathbf{x}_t^T Q \mathbf{x}_t + \mathbf{u}_t^T R \mathbf{u}_t)] \tag{A.3}$$

System matrices

The system matrices for equation 1 can be constructed using equations 2, 0.1,6 and 8. To do so we also need to derive an explicit form for the vehicle linear acceleration (eq 8). We do this by computing it via finite difference,

$$a_{t+1}^v = \frac{1}{\Delta_t}(v_{t+1}^v - v_t^v) \tag{A.4}$$

$$a_{t+1}^v = \frac{1}{\Delta_t}(\lambda(p_t^w + \Delta_t v_t^w) + \epsilon_t^{v^v} - v_t^v) \tag{A.5}$$

$$a_{t+1}^v = \frac{\lambda}{\Delta_t}p_t^w + \frac{1}{\Delta_t}\lambda\Delta_t v_t^w + \frac{1}{\Delta_t}\epsilon_t^{v^v} - \frac{1}{\Delta_t}v_t^v \tag{A.6}$$

$$a_{t+1}^v = \frac{\lambda}{\Delta_t}p_t^w + \lambda v_t^w + \frac{1}{\Delta_t}\epsilon_t^{v^v} - \frac{1}{\Delta_t}v_t^v \tag{A.7}$$

We can now combine all the equations together to give us our matrix representation of the system,

$$A = \begin{bmatrix} 1 & \Delta_t & 0 & 0 & 0 & 0 & 0 \\ 0 & 0 & 0 & \lambda & \lambda\Delta_t & 0 & 0 \\ 0 & -\frac{1}{\Delta_t} & 0 & \frac{\lambda}{\Delta_t} & \lambda & 0 & 0 \\ 0 & 0 & 0 & 1 & \Delta_t & 0 & 0 \\ 0 & 0 & 0 & 0 & 1 & \frac{\Delta_t}{m} & 0 \\ 0 & 0 & 0 & 0 & 0 & (1-\frac{\Delta_t}{\tau_{nm}}) & \frac{\Delta_t}{\tau_{nm}} \\ 0 & 0 & 0 & 0 & 0 & 0 & (1-\frac{\Delta_t}{\tau_{nm}}) \end{bmatrix}, \mathbf{x}_t = \begin{bmatrix} p_t^v \\ v_t^v \\ a_t^v \\ p_t^w \\ v_t^w \\ f_t^w \\ g_t^w \end{bmatrix}, B = \begin{bmatrix} 0 \\ 0 \\ 0 \\ 0 \\ 0 \\ 0 \\ \frac{\Delta_t}{\tau_{nm}} \end{bmatrix},$$

$$V = \sigma_m B, V_\epsilon = \begin{bmatrix} 0 & 0 & 0 & 0 & 0 & 0 & 0 \\ 0 & 1 & 0 & 0 & 0 & 0 & 0 \\ 0 & \frac{1}{\Delta_t} & 0 & 0 & 0 & 0 & 0 \\ 0 & 0 & 0 & 0 & 0 & 0 & 0 \\ 0 & 0 & 0 & 0 & 0 & 0 & 0 \\ 0 & 0 & 0 & 0 & 0 & 0 & 0 \\ 0 & 0 & 0 & 0 & 0 & 0 & 0 \end{bmatrix} R = \begin{bmatrix} r \end{bmatrix} Q = \begin{bmatrix} 1 & 0 & 0 & 0 & 0 & 0 & 0 \\ 0 & 0 & 0 & 0 & 0 & 0 & 0 \\ 0 & 0 & 0 & 0 & 0 & 0 & 0 \\ 0 & 0 & 0 & 0 & 0 & 0 & 0 \\ 0 & 0 & 0 & 0 & 0 & 0 & 0 \\ 0 & 0 & 0 & 0 & 0 & 0 & 0 \\ 0 & 0 & 0 & 0 & 0 & 0 & 0 \end{bmatrix}$$

Similarly we can rewrite our sensory feedback (eq 9,11 and 12) in the form of Eq 15. When there is no delay we can write the observations as,

$$G = \mathrm{diag}([\sigma^{p^v}, \sigma^{v^v}, \sigma^{a^v}, \sigma^{p^w}, \sigma^{v^w}, \sigma^{f^w}]) = \sigma_s \mathrm{diag}([0.02, 0.2, 0.5, 2, 20, 100])$$

$$H = \begin{bmatrix} 1 & 0 & 0 & 0 & 0 & 0 & 0 \\ 0 & 1 & 0 & 0 & 0 & 0 & 0 \\ 0 & 0 & 1 & 0 & 0 & 0 & 0 \\ 0 & 0 & 0 & 1 & 0 & 0 & 0 \\ 0 & 0 & 0 & 0 & 1 & 0 & 0 \\ 0 & 0 & 0 & 0 & 0 & 1 & 0 \end{bmatrix},$$

We choose to use this G matrix as it scales the noise to the units of the observable variables, position, velocity and acceleration [2]. To incorporate the different sensory delays into our model, we need access to the prior states of the system at certain points in time (e.g at $t - \delta_{t_{vis}}$). One way to do this is to augment the state space to contain the states at previous points in time [3,4]. The maximum number of previous time steps we need to augment the state space by is equal to the largest delay over the different observations $\delta_{\max}$. This yields an LQG system with the following new state space,

$$\text{System } : \overline{\mathbf{x}}_{t+1} = \begin{bmatrix} A & \cdots & 0 & 0 \\ I & \dots & 0 & 0 \\ \vdots & \ddots & \vdots & \vdots \\ 0 & \cdots & I & 0 \end{bmatrix} \begin{bmatrix} \mathbf{x}_t \\ \mathbf{x}_{t-1} \\ \vdots \\ \mathbf{x}_{t-\delta_{\max}} \end{bmatrix} + \begin{bmatrix} B \\ 0 \\ \vdots \\ 0 \end{bmatrix} \mathbf{u}_t + \begin{bmatrix} V \\ 0 \\ \vdots \\ 0 \end{bmatrix} \boldsymbol{\eta}_t + \begin{bmatrix} V_\epsilon \\ 0 \\ \vdots \\ 0 \end{bmatrix} \boldsymbol{\epsilon}_t \quad \text{(A.8)}$$

$$\overline{\mathbf{x}}_{t+1} = \overline{A}\overline{\mathbf{x}}_t + \overline{B}\mathbf{u}_t + \overline{V}\boldsymbol{\eta}_t + \overline{V}_\epsilon \overline{\boldsymbol{\epsilon}}_t \quad \text{(A.9)}$$

Given this new augmented state space we can define the necessary observation matrix by retrieving the state from the appropriate previous states. This can be constructed from the rows of the original observation matrix H_i, alongside the corresponding delays for each row, which here yields,

$$\text{Feedback } : \mathbf{y}_t = \overline{H}\overline{\mathbf{x}}_t + G\boldsymbol{\omega}_t \quad \text{(A.10)}$$

$$\mathbf{y}_t = \begin{bmatrix} y_t^{p^v} \\ y_t^{v^v} \\ y_t^{a^v} \\ y_t^{p^w} \\ y_t^{v^w} \\ y_t^{f^w} \end{bmatrix} = \begin{bmatrix} \underbrace{0 \quad 0 \quad 0 \quad \cdots}_{\delta t_{vis}} \; H_1 \\ \underbrace{0 \quad 0 \quad 0 \quad \cdots}_{\delta t_{vis}} \; H_2 \\ \underbrace{0 \quad 0 \quad \cdots}_{\delta t_{ves}} \; H_3 \quad 0 \\ H_4 \quad 0 \quad \cdots \quad 0 \quad 0 \\ H_5 \quad 0 \quad \cdots \quad 0 \quad 0 \\ H_6 \quad 0 \quad \cdots \quad 0 \quad 0 \end{bmatrix} \begin{bmatrix} \mathbf{x}_t \\ \mathbf{x}_{t-1} \\ \mathbf{x}_{t-2} \\ \vdots \\ \mathbf{x}_{t-\delta_{\max}} \end{bmatrix} + G\boldsymbol{\omega}_t \quad \text{(A.11)}$$

Note that, zeros refer to vectors of appropriate size, entailing that position and velocity are read out with visual delay, and acceleration is read out with the vestibular delay. Similarly, we can define our cost in terms of this new augmented system,

$$\text{Cost}\ : J = \frac{1}{2}\mathbb{E}[\sum_{t=0}^{\infty}(\overline{\mathbf{x}}_t^T\overline{Q}\overline{\mathbf{x}}_t + \mathbf{u}_t^T R\mathbf{u}_t)] \tag{A.12}$$

$$= \frac{1}{2}\mathbb{E}[\sum_{t=0}^{\infty}(\begin{bmatrix}\mathbf{x}_t & \mathbf{x}_{t-1} & \cdots & \mathbf{x}_{t-\delta_{\max}}\end{bmatrix}\begin{bmatrix}Q & 0 & \cdots & 0\\ 0 & 0 & \cdots & 0\\ \vdots & \vdots & \ddots & \vdots\\ 0 & 0 & \cdots & 0\end{bmatrix}\begin{bmatrix}\mathbf{x}_t\\ \mathbf{x}_{t-1}\\ \vdots\\ \mathbf{x}_{t-\delta_{\max}}\end{bmatrix} + \mathbf{u}_t^T R\mathbf{u}_t)] \tag{A.13}$$

Optimal policy

An ideal participant utilises a policy $\mathbf{u}_t \sim \pi_t(\mathbf{y}_t)$ to map from the incoming observations to actions such that some performance metric is optimized. In general, this can be stochastic function and thus $\mathbf{u}_t$ can be a distribution, but in our model it is a deterministic function of the observations. The central problem for the participant is to determine what policy to use for our task. We assume, for tractability, that the participant treats the unknown perturbation as a Gaussian random variable, which allows us to deal with the delayed augmented system as a standard LQG system. The optimal policy (with respect to the participant's knowledge) can be derived analytically via dynamic programming, which results in a combination of a optimal linear estimator (a Kalman filter) and a linear affine controller (the optimal LQR controller). For a detailed proof see [1].

Estimation

Given the assumption of an LQG system, the optimal estimator is a Kalman filter with matrices matched to the true system. Given that we utilize an external perturbation in our model, the statistics and structure (e.g white vs band-pass) of the perturbation must also be learned by the participant. Thus, our participants must consider three distinct variance terms (in terms of the system) which impact the covariance of the Kalman filter: the motor noise (σ_m) of the participant, the participant's internal estimate of the perturbation variance (σ_ϵ), and the noise which corrupts the participant's estimate σ_ξ [2,3]. In this case the optimal solution is to incorporate these distinct noise terms into the estimation process of the Kalman filter (effectively as an additional process noise term). The 'process noise' covariance (in the sense that it is analogous to process noise in the standard Kalman filter) becomes,

$$\Sigma_{pred} = \sigma_m^2 BB^T + \sigma_\epsilon^2 V_\epsilon V_\epsilon^T + \text{diag}(\sigma_\xi^2) \tag{A.14}$$

Note, we use the same V_ϵ as in the true system V, which represents participants knowledge of the correlation between velocity and acceleration. The estimate noise covariance is a diagonal matrix the size of the augemented space, thus, implying that noise is added to the estimate at each time step.

Combing this covariance with an internal model of the necessary matrices (A, B, H, G), the participant can compute the optimal estimate via Eq (19). In order to do this, the participant needs to determine the optimal Kalman gain, which is the gain that minimizes the estimation error between the filter estimate and true state. This can be precomputed iteratively by the following Algebraic Ricatti equation,

$$P_{t+1} = AP_tA^T - AP_tH^T(HP_tH^T + GG^T)^{-1}HP_tA^T + \Sigma_{pred} \tag{A.15}$$

$$K_t = P_tH^T(HP_tH^T + GG^T)^{-1} \tag{A.16}$$

Fixed variables	Description	Value
m	Mass (kg)	1
σ_m	Motor noise (N/s)	5
τ_{nm}	Muscular time constant (ms)	40
σ_ϵ	Assumed perturbation gain (unitless)	0.25
σ_ξ	Estimate noise gain (unitless)	$8e^{-7}$
Δ_t	Time step size (ms)	0.017
λ	Wheel angle to vehicle velocity (m/s per deg)	0.008
$\delta_{t_{vis}}$	Visual delay (time steps)	7
$\delta_{t_{ves}}$	Vestibular delay (time steps)	1
$\delta_{t_{prop}}$	Proprioceptive delay (time steps)	0
**Fitted variables**		
r^e_{vis}	Visual control cost exponent	-7.56 ± 0.11
r^e_{visuo}	Visuoinertial control cost exponent	-8.36 ± 0.11
σ_s	Sensory noise gain (unitless)	0.19 ± 0.02

Table A. Table of parameters used for primary model predictions, including notation, description and value. Fitted values given as the participant average ± sem.

All our our matrices $(A, B, H, G, \Sigma_{pred})$ are time invariant and thus P_t approaches a fixed steady state. The final converged value of P_t is then used in Eq (19).

Control

As we are considering a classic LQG system, the optimal controller and optimal estimator are independent. With the optimal feedback gain given by [1],

$$S_t = A^T S_{t+1} A - A^T S_{t+1} B (B^T S_{t+1} B + R)^{-1} B^T S_{t+1} A + Q \tag{A.17}$$

$$L_t = (B^T S_{t+1} B + R)^{-1} B^T S_{t+1} A \tag{A.18}$$

Given our cost function and matrices are also temporally invariant, this converges to a stationary gain L which we use in our controller (eq 20).

Model frequency response properties

In our primary analysis both the participants' data and our model show an attenuation at higher amplitudes which is indicative of a low pass filter. Where does this effect come from? The frequency response of our model is dictated by multiple components from the plant itself to the controller and estimator. Does the low pass response originate from a single component? Or a combination of them? One method to asses this is to evaluate the frequency response of simplified versions of the model. Here we explore the frequency response of a few reduced models to better understand the frequency response of the full model. To start we simplify the model by making it fully observable (so no estimator is needed), removing all noise outside the perturbation, and eliminating low pass muscle filter from the plant. Thus, in this case, the participant is able to control force directly. Under these changes our model equations become,

$$p^v_{t+1} = p^v_t + \Delta_t v^v_t \tag{A.19}$$
$$v^v_{t+1} = \lambda(p^w_t + \Delta_t v^w_t) + \epsilon^{v^v}_t \tag{A.20}$$
$$p^w_{t+1} = p^w_t + \Delta_t v^w_t \tag{A.21}$$
$$v^w_{t+1} = v^w_t + \frac{\Delta_t}{m} u^w_t \tag{A.22}$$

We start by rewriting this system to eliminate v^v_{t+1} by substituting it into p^v_{t+1},

$$p^v_{t+1} = p^v_t + \Delta_t \lambda p^w_t + \Delta_t \epsilon^{v^v}_{t-1} \tag{A.23}$$
$$p^w_{t+1} = p^w_t + \Delta_t v^w_t \tag{A.24}$$
$$v^w_{t+1} = v^w_t + \frac{\Delta_t}{m} f^w_t \tag{A.25}$$

This can be re written in the form of Eq 1,

$$\mathbf{x}_{t+1} = \underbrace{\begin{bmatrix} 1 & \Delta_t\lambda & 0 \\ 0 & 1 & \Delta_t \\ 0 & 0 & 1 \end{bmatrix}}_{A} \underbrace{\begin{bmatrix} p^v_t \\ p^w_t \\ v^w_t \end{bmatrix}}_{\mathbf{x}_t} + \underbrace{\begin{bmatrix} 0 \\ 0 \\ \frac{\Delta_t}{m} \end{bmatrix}}_{B} \underbrace{u^w_t}_{\mathbf{u}_t} + \underbrace{\begin{bmatrix} \Delta_t \\ 0 \\ 0 \end{bmatrix}}_{V_\epsilon} \underbrace{\epsilon^{v^v}_{t-1}}_{\boldsymbol{\epsilon}_t}$$
$$\mathbf{u}_t = -L\mathbf{x}_t$$

We are interested in assessing how the perturbation influences the subjects currently commanded sled velocity $\lambda(p^w_t)$ (as this is used to compute gain in the main text). To do so we rewrite this system such that the input $\mathbf{u}_t$ is the perturbation, the commanded sled velocity is the output, and the closed loop (e.g controlled) dynamics are the system dynamics.

$$\mathbf{x}_{t+1} = \underbrace{(A - BL)}_{A_{clo}} \mathbf{x}_t + \underbrace{\begin{bmatrix} \Delta_t \\ 0 \\ 0 \end{bmatrix}}_{B_{clo}} \mathbf{u}_t$$
$$\mathbf{u}_t = \boldsymbol{\epsilon}_t, y = \begin{bmatrix} 0 & \lambda & 0 \end{bmatrix} \mathbf{x}_t$$

We can then compute the appropriate transfer function of this system and generate a corresponding bode plot. The corresponding bode plot is shown in S1 Fig. Compared to the primary model predictions in Fig 6, the gain plot is comparable to the predictions of the primary model, but we see what the phase difference is generally lower in the direct force model than in the primary model predictions. In order to asses the influence of the low-pass neuromuscular filter (but without the estimator) we add it into this simplified model. This yields a new linear system,

$$\mathbf{x}_{t+1} = \underbrace{\begin{bmatrix} 1 & \Delta_t\lambda & 0 & 0 & 0 \\ 0 & 1 & \Delta_t & 0 & 0 \\ 0 & 0 & 1 & \frac{\Delta_t}{m} & 0 \\ 0 & 0 & 0 & 1 - \frac{\Delta_t}{\tau_{nm}} & \frac{\Delta_t}{\tau_{nm}} \\ 0 & 0 & 0 & 0 & 1 - \frac{\Delta_t}{\tau_{nm}} \end{bmatrix}}_{A} \underbrace{\begin{bmatrix} p^v_t \\ p^w_t \\ v^w_t \\ f^w_t \\ g^w_t \end{bmatrix}}_{\mathbf{x}_t} + \underbrace{\begin{bmatrix} 0 \\ 0 \\ 0 \\ 0 \\ \frac{\Delta_t}{\tau_{nm}} \end{bmatrix}}_{B} \underbrace{u^w_t}_{\mathbf{u}_t} + \underbrace{\begin{bmatrix} \Delta_t \\ 0 \\ 0 \\ 0 \\ 0 \end{bmatrix}}_{V_\epsilon} \underbrace{\epsilon^{v^v}_{t-1}}_{\boldsymbol{\epsilon}_t}$$
$$\mathbf{u}_t = -L\mathbf{x}_t$$

The corresponding bode plot for this model is given in S1 Fig. When the neuromuscular low-pass filter is added back, we see that the phase plot becomes close to that of the motion condition. This suggests in our motion condition most of the low pass filter nature, in term of gain, comes from the controller itself, and to a lesser degree the neuromuscular low-pass, and the estimator. By contrast, in the stationary condition, the additional phase effect is due to the estimator increasing the phase difference.

References

1. Stengel RF. Optimal control and estimation. Dover books on advanced mathematics. New York: Dover Publications; 1994.

2. Todorov E. Stochastic Optimal Control and Estimation Methods Adapted to the Noise Characteristics of the Sensorimotor System. Neural Computation. 2005;17(5):1084–1108. doi:10.1162/0899766053491887.

3. Crevecoeur F, Munoz DP, Scott SH. Dynamic Multisensory Integration: Somatosensory Speed Trumps Visual Accuracy during Feedback Control. The Journal of Neuroscience. 2016;36(33):8598–8611. doi:10.1523/JNEUROSCI.0184-16.2016.

4. Todorov E, Jordan MI. Optimal feedback control as a theory of motor coordination. Nature Neuroscience. 2002;5(11):1226–1235. doi:10.1038/nn963.
