# Supplementary material for: Visuoinertial and visual feedback in online steering control
Source: PLoS Comput Biol. 2025 Aug 11;21(8):e1012659. doi: 10.1371/journal.pcbi.1012659 (PMC12360656; doi:10.1371/journal.pcbi.1012659)
Supplement: Attachment — Submitted filename: reviews_reply.pdf [file pcbi.1012659.s003.pdf]

Response to reviewers (manuscript PCOMPBIOL-D-24-02019)

We thank the three reviewers for their careful evaluations. Below, you will find each of their comments, followed by our reply in blue. The figure and page/line numbers refer to the figures and lines in the tracked-changes version of the revised manuscript.

Reviewer #1: In this manuscript, Liu and colleagues present an innovative closed-loop control task in which participants stabilize the position of a simulated car on a road. This task is implemented using a motorized sled under two conditions: visual-only and visuo-vestibular. The authors find that participants' ability to control their trajectories under perturbations exhibits a low-pass dynamic, more pronounced in the visual-only condition than in the visuo-vestibular condition. This result aligns with prior knowledge that the vestibular system is more accurate than vision at high frequencies.

The authors also propose a detailed control model and attempt to fit it to the data. However, I have significant reservations about this model. Their attempt to fit both conditions with a single set of parameters ("Model Fit 1") is largely unsuccessful, as this approach fails to reproduce the gain differences and only marginally explains the phase differences between the two conditions (Fig. 6A). They achieve better results by allowing the model to use two separate sets of parameters (Fig. 6B), but the resulting best-fitting parameters differ dramatically, especially in the control parameters. This suggests that the model cannot adequately capture the differences between the visual-only and visuo-vestibular conditions. This is surprising, given the extensive body of work on dynamic visuo-vestibular integration over the past decades (e.g., Raphan/Cohen, Merfeld, Laurens, and Karmali).

Due to these limitations, I do not believe the modeling component of the manuscript is ready for publication. While the model is grounded in sound hypotheses and the established formalism of optimal control, it is puzzling that it fails to reproduce basic phenomena, such as the vestibular system's contribution to high-frequency self-motion perception. Furthermore, the approach used for fitting "Model 2" raises significant concerns. Addressing these issues will require substantial additional work.

We appreciate the reviewer's thoughtful evaluation of the model fits. As we acknowledged in our original manuscript, our initial model fits using a brute-force grid search technique were not optimal. In response to the reviewer's suggestion, we have re-examined our model fitting procedure, drawing inspiration from Nash and Cole (2019). Specifically, whereas we previously fit our model in the frequency domain, we now fit the model parameters to the steering response in the time domain, using an adaptive search algorithm. This approach provided realistic values of visual noise (0.5 cm position noise) and vestibular noise (10

cm/s2), consistent with prior literature. By applying this revised fitting procedure, we achieved very good fits to the data, especially in the visual-only condition. The fits were good for gain but somewhat less accurate for the visuo-inertial condition, where the model tended to overestimate phase compared to the participants' actual performance. In the revised manuscript, we have updated the model fitting and simulation sections in the Methods (including Fig. 3), as well as the model fits presented in the Results (Figs. 4 and 6A). Additionally, we now illustrate how well the model accounts for the data in the time domain (Fig. 6B). We hope these revisions address the reviewer's main concerns.

Major Comments

Model Fitting:

As noted, "Model Fit 1" fails to explain the differences between visual-only and visuo-vestibular conditions. In "Model Fit 2," the control cost parameter r is effectively set to zero in the visuo-vestibular condition (and to 1 in the visual condition). In essence, this approach essentially differences in performance across sensory contexts to changes in the control policy, which is illogical, and fails to capture the underlying sensory differences.

Agreed. This comment has been resolved with the new fitting approach, without relying on differences in sensory parameters.

In view of this, the claim in the abstract, "Using computational modeling, we demonstrate that this enhanced performance is partially due to the shorter delay of the vestibular modality," appears to be unsupported. In fact, the last paragraph on page 16 of the Discussion seems to explicitly contradict this statement.

We have removed the reference to these two separate model fits, making this comment no longer applicable.

The authors also use a parameter σs, which scales all sensory noise collectively. This parameter raises several concerns:

(1) It needs to be clearly defined in the text, as the only explicit explanation appears in the equation on page 18 (σs.diag([0.02,0.2,1,0.02,0.2,1])).

Done, we added to the respective text: "G involves a scaling constant σs to scale noise levels without changing their relative magnitudes (see Appendix for details). See page 8, line 267.

(2) It is illogical that all sensory noise, including vision and proprioception, should change by a factor of 9 in the visual-only condition. Moreover, it is odd that noise levels are lower in the visual-only condition, where human performance is worse.

See reply above. With the new model fitting, this comment is no longer applicable.

(3) The default value of vestibular noise (σ_{va}=1m/s2) appears quite high, which may explain why the model struggles to explain the vestibular contribution to steering.

Agreed. The new model settles on more realistic noise values to account for the data. That is vestibular noise now leveled at 0.1 m/s2 (which is consistent with Guedry 1974; MacNeilage et al. 2010).

These points raise concerns about the grid search procedure, where all parameters were scaled by a single factor. It is possible that an insufficiently explored parameter space or an unrealistic parameter combination is driving the observed results.
Based on this, I would recommend that the authors revisit the model design and/or fitting process. While the model shows promise, the current conclusions, based on the fitting approach in Model 2, cannot be accepted.

We are grateful to the reviewer for their valuable insights which has significantly improved our fitting procedure. We have addressed many of the critical concerns and removed Model 2.

Minor Comments
Page 1: The statement, "Although studies on multisensory integration indicate that sensory inputs are combined optimally based on their precision, these findings have largely been derived from static experimental settings," overlooks previous work on Kalman filters and Bayesian estimators for dynamic self-motion perception (visual and vestibular).

We acknowledge that the sentence may overly narrow the focus of the summary and have therefore removed it in the revision.

Fig. 1D: The labels on the time axis are all 0.

We apologize for this mistake, which has now been corrected.

Page 5, second paragraph: I imagine that the sled limits (0.4 m left, 0.6 m right) simulate the driver's offset position relative to the car's center?

We apologize for the confusion, which has been resolved by removing the respective sentence. There was no offset: the task for the participant was to keep the car on the road, while sled motion was perturbed within the 0.9 m range of the track length.

Page 7: The sentence, "Due to this choice, our perturbation is manually mapped to the acceleration state in the true system via $V\epsilon$" is unclear.

We explicitly had to incorporate the acceleration perturbation into the model, using a separate matrix. Omitting this step would result in the model only detecting changes in velocity and position, thereby overlooking acceleration, which would render the model to be inadequate. We added the following text to the revised manuscript: “As a result of this choice, we had to explicitly incorporate the acceleration perturbations into the model, using a separate matrix V_ϵ . This approach enables the model to detect changes not only in velocity and position, but also in acceleration.” See page 7, line 239.

Based on equation (5), the force exerted on the wheel (fw) is equivalent to the acceleration of the control signal. While this is clear based on the equations, the authors should explain this explicitly in the text. Otherwise, readers may find the interchangeable use of “steering force” and “acceleration” confusing.

Done. We added to the text (page 7, line 248): Thus, the force exerted on the wheel (fw) is equivalent to the acceleration component of the control signal.

Figure 3: This figure is critical for understanding the model's predictions, but is very difficult to read. Please add legends directly within the panels.

Done. We have revised the figure so that each panel now displays the predictions for both conditions, allowing for easier comparison. All predictions are based on the new model simulations.

Page 11: The statement, “Combined, this indicates that the primary factor influencing the frequency response characteristics of the model is the controller, while the neuromuscular system and the estimator play a lesser role,” might be influenced by the use of high control costs in all simulations. Consider setting the control cost to zero, and then explore the effects of sensory noise and delay (for the visual and vestibular modality independently).

This comment has been addressed with the updated model fits. Additionally, we note that this point pertains to the influence of the neuromuscular system, as demonstrated in the appendix, which shows that these characteristics are less significant compared to the controller itself.

Fig. 4B: The Y-axis labels are inconsistent, with intervals alternating between 0.02 and 0.03.
Table 1: There is a typo in the description of the parameter λ (vehicle).

Apologies, this was remnant of a rounding approach, which has now been corrected. We have also corrected the typo.

Reviewer #2: The authors study multisensory integration in a dynamic environment requiring to take into account time-varying sensory signals and, therefore, their respective delays. Participants were asked to counteract lateral perturbations to keep a virtual car on the road. The perturbations consisted of a pseudo-random sum of sines added to the speed signal. Two conditions were tested: visual only or visuo-inertial, in which the participants' seats were moved laterally, adding vestibular information to the sensory feedback on the perturbations. It is shown, based on gain and phase, that the model with multimodal estimates performs better and reproduces the participants' behaviour qualitatively.

I found the manuscript very well written and organized. It makes an interesting and important contribution to the field. In my opinion, the paper could be published as is. I have only a number of questions or suggestions to clarify the text and explore alternative hypotheses:

1- I fully agree with the author's basic premise that standard models of multisensory integration that only consider variance do not generalise to dynamic conditions because dynamics and delays matter. I believe that the authors demonstrate successfully that their model captures some aspects of behaviour, which supports the hypothesis that vision and vestibular signals are combined dynamically, but was there any evidence against a non-dynamic weighting scheme? In Crevecoeur et al. (2016, cited), the constant saccadic reaction time with or without vision was not compatible with linear combination of vision and proprioception. Could the authors explore if or how participant's behaviour was incompatible with static multisensory integration?

This hypothesis is challenging to test given our current study design and the data we have collected. For instance, unlike Crevecoeur et al. (2016), we lack time-aligned measurements and predictions for saccades. We believe that direct experimental paradigms to explicitly test this distinction in online steering control would be valuable for future research

2- I found that Figure 3 was difficult to interpret: what is the most important piece of information? Is it that the orange traces in the bottom right figure are different from the blue ones, showing the effect of vestibular input? If I interpret correctly, the benefit of vestibular was to reduce the phase of the response, meaning that the estimate was closer to the state, enabling "less-out-of-phase". If this is visible on traces in the temporal domain, adding such traces may help the reader navigate through these plots.

The reviewer's interpretation of phase is correct. We added the following sentence to the revised text on page 10, line 343. "Thus, vestibular input helps decrease the phase lag in

the response, allowing the estimate to more closely match the actual state and resulting in a response that is less out of phase." We also added legends directly within the panels to enhance the clarity of the model's predictions. Because we use a multi-sine perturbation, analyzing phase differences directly from temporal traces (as shown in Fig. 4) is challenging. Instead, it is more effective to employ system identification techniques for this purpose.

3- I had a question regarding the treatment of the perturbation signal (epsilon_t), which was treated as a noise term. Alternatively, it could be considered as constant or even dynamical input signal. In particular, if participants noticed that perturbation followed sinusoidal traces (at least for some frequencies), this knowledge can be added to the model. Can the authors comment on that? What would be the consequence of adding sinusoidal dynamics to epsilon in the model? Can the authors discuss the impact of considering epsilon as noise?

Our approach of using the sum-of-sines method to generate a perturbation that resembles white noise in the time domain is a well-established technique in signal processing and modelling. This approach is supported by its characteristics of flat power spectral densities, zero mean, and constant variance. Consequently, we deemed it appropriate to represent our perturbation as white noise within the model, considering it a valid and practical decision. Yet, we do certainly not claim that participants are unable to detect periodicity in the perturbation and adapt their control strategies accordingly.

The optimized fitting procedure (in response to Reviewer 1) revealed that the model could not fully account for the phase observations in the visuo-inertial condition, as these were smaller than predicted. To investigate whether participants adapt their control strategies by estimating the perturbation, we extended the model as suggested by the reviewer. Specifically, we treated the perturbation as a state (an external velocity which gets added to the sled velocity), which can change over time (e.g. it has a velocity state the observer can also estimate). This adjustment allows the subject to now estimate the perturbation state allowing them to incorporate it into the controller and estimator. The figure below shows that this modification allows the model to more accurately capture the observed phase behavior in the visuo-inertial condition. However, this improvement appears to come at the expense of a mismatch in gain. For this reason, we have decided not to include this revised version of the model in the updated manuscript. Of course, it is possible an alternative perturbation estimation model could account for our data, so we have added a discussion section to highlight this for future work.

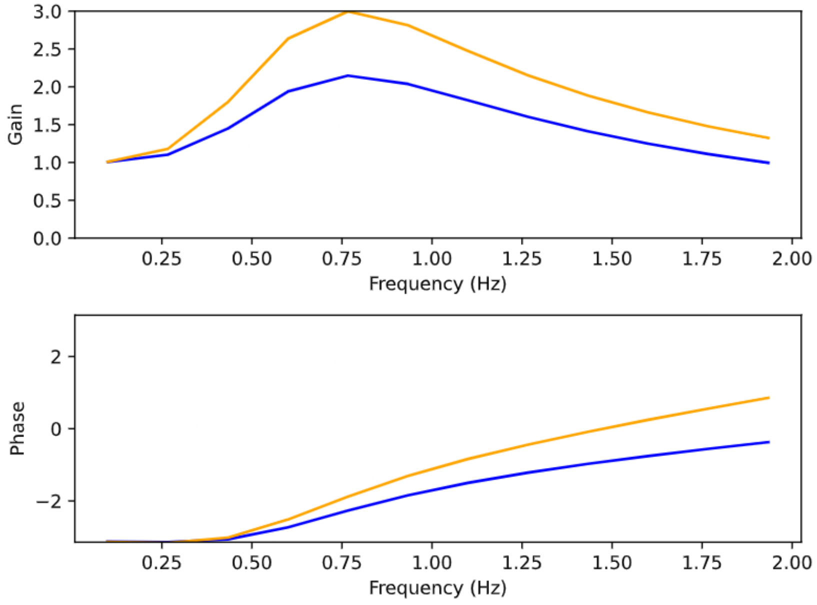

4- Equation 19: I was surprised to see that the prediction noise (xi_t) appears in the term multiplied by the Kalman gain. If the controller/estimator structure knows this noise term, it can be subtracted and removed. Typically, prediction noise does not impact the terms dependent on Kalman gains (see Eqns. 5.1 in Todorov, 2005, ref 33). If there is a genuine argument for the double appearance of xi_t, it should be better justified.

For the model we utilized a filtering rather than predictive solution. In the predictive solutions (Todorov, 2005), the reviewer is correct that the noise term would not appear in the K term. For the filtering solution (Crevecour et al, 2011), it does appear as it is added to the prediction directly (and is treated as an unknown noise so cannot be removed). We have changed the text to highlight the two-step procedure (prediction then update) to make this more apparent.

5- Figure 5: the arbitrary offset in the ordinate axis (0.08) produces an inflated visual effect size. Pls consider alternative data representation.

Done. We have redrawn the figure using standard axes.

6- Page 8: it is written that the vestibular feedback delay was 10ms, whereas the appendix has an observability matrix H that sits at an index larger than the proprioceptive delay (Eqn. A.11). Could the authors check/clarify?

Since the proprioceptive system of the arms does not deliver direct information regarding lateral body translation, we presumed that the position, velocity, and force related to steering the wheel were accessible without any delay.

7- Just before Eqn. 12 it is written that the proprioceptive delay was 0ms, however this seems inconsistent with the physics of the system. In the condition including lateral displacement, the proprioceptive system will also convey information about motion. This seemd aslo inconsistent with Eqn. A.11, which shows delta_prop > 0. Pls clarify.

See previous comment. It is important not to confuse proprioception of the arms with somatosensory signals that inform about body translation. This has been emphasized in the revised text (page 8, line 253). In relation to the motion, we consider the somatosensory and vestibular together in the visuo-inertial condition and approximate their delay as the delay of the vestibular system. Regarding eq A.11, we indeed assumed that the delay was 0 we have updated A.11 to show this.

8- Eqn. A.11: Pls consider adding indices to the "zeros" to show that these are block matrices, and the product with the time series of state vector is block-wise.

We like to emphasize that the 0s are not block matrices, they are vectors, as each Hi are rows from the original matrix, not the entire matrix. Confusion probably stems from the reviewers' conceptualization of the H matrix, where each modality has a read out of position, velocity and acceleration. In our case, position and velocity are read out with visual delay, and acceleration is read out with the vestibular delay. We have clarified our writing; page 19, line 611.

Reviewer #3: The study addresses an important question, which is multisensory integration for active, dynamic behaviors. The experimental methods, modeling and analysis are well-developed and clearly presented with appropriate care taken to identify assumptions and caveats. I have only minor comments/suggestions.

Suggest line numbers for easier referencing in future

Introduction, line 3: I'm not familiar with this designation of optic flow processing pathways as the 'optokinetic system'. Optokinetic is most often used in the context of reflexive eye movements and may be confused with specific pathways involved in generating those reflexes.

We removed any reference to 'optokinetic' as well as rewrote the respective sentence as follows: "Our perception of self-motion relies mainly on visual cues—like optic flow—as well as inertial sensory information from the vestibular system, and possibly the somatosensory system as well." See page 2, line 45.

Figure 1. Based on the depiction here, the visual stimulus does not involve optic flow. Instead, this is more of an object motion stimulus, i.e. how is the car moving relative to the static visual background. This is much less ecological than if a first-person view of the road had been used. Some words to acknowledge this observation (and perhaps a justification of this choice) are warranted given the large literature on visual-vestibular integration most often uses optic flow. This should also be acknowledged in the interpretation offered in the discussion section. The observed importance/effect of visual latency may have also been observed if optic flow has been used, so the result may generalize. Nevertheless, I think the non-ecological nature of the stimulus should be acknowledged.

We would like to note that when participants are in motion, the entire visual display—including the road and surrounding environment—creates optic flow on the participants' retinas. However, we agree with the reviewer that our stimulus may not be the most ecologically valid, a limitation we have acknowledged in the revised text. On page 6, line 210, we state: "While the task did not employ a first-person view of the road—making it perhaps less ecologically valid than actual steering—correction for perturbations relies on visual and inertial cues, which are inherently noisy and subject to time delays".

What is the system latency for the impact of a steering command on the movement of the car and/or platform? Is this latency accounted for in the model? If not, what are possible consequences?

The reviewer asks about the transfer function of the sled, which is close to unity for frequencies up to 2.5 Hz. We have added this information to the revised text: page 6, line 223.

Page 6, bottom: 'Hence, correcting for the perturbation relies on feedback from the visual and/or mostly the vestibular system' Why use the word 'mostly' here? I realize that results suggest vestibular cues provide an advantage, but that observation does not belong here in the methods.

We intended to convey that inertial cues are primarily, though not exclusively, detected by the vestibular system; for instance, somatosensory cues may also contribute. Accordingly, we have revised the part of sentence as follows: ".. correction for perturbations relies on visual and inertial cues, which are inherently noisy and subject to time delays. See page 6, line 223.

Page9, last sentence before Model Simulations: the further dyanmics… it would be worth expanding this observation and explaining in detail whey these parameters are the ones that are expected ot impact dynamics…

These interactions are specified by the model, and its corresponding equations, as detailed in the main text and appendix. Revised figure 3 illustrates these interactions more explicitly; we have therefore included a reference to this figure to highlight the subsequent dynamics.

Page 10/11: Much of the section on model simulations reads like a results section; suggest moving this text and figures to the results.

Unless insisted otherwise by the reviewer or editor, we believe that maintaining a clear distinction between model development/simulations and the results provides a more coherent narrative.

Figure 4: I think that the bars in figure 4 B are superimposed (i.e. the yellow is continuous behind the blue bar), but these could easily be confused for a stacked bar plot. It is worth clarifying.

We clarified the figure, showing that the bars are superimposed.
